# Cost and Clinical Benefits Associated with Oncotype DX® Test in Patients with Early-Stage HR+/HER2- Node-Negative Breast Cancer in the Netherlands

**DOI:** 10.1155/2022/5909724

**Published:** 2022-09-22

**Authors:** Felix E. de Jongh, Reva Efe, Kirsten H. Herrmann, Jelle A. Spoorendonk

**Affiliations:** ^1^Breast Cancer Center South Holland South, Ikazia Ziekenhuis Rotterdam, Montessoriweg 1, 3083 AN, Rotterdam, Netherlands; ^2^OPEN Health, Marten Meesweg 107, 3068 AV, Rotterdam, Netherlands; ^3^Exact Sciences Deutschland GmbH, 51103 Cologne, Erna-Scheffler-Str. 1a, Germany

## Abstract

**Objectives:**

Patients with early-stage HR+/HER2- N0 breast cancer may receive adjuvant chemotherapy in combination with surgery. However, chemotherapy does not always lead to improved survival and incurs high healthcare costs and increased adverse events. To support decision-making regarding adjuvant chemotherapy, genomic profile testing performed with tests such as the Oncotype DX® test can help healthcare practitioners decide whether chemotherapy provides any benefit to these patients. As such, a cost-consequence model was developed with the aim to estimate the economic impact of using different gene expression tests or no testing, in patients with node-negative early-stage breast cancer.

**Methods:**

A cost-consequence model was developed to estimate the economic impact of three different scenarios in the Dutch setting: (1) Oncotype DX® test, (2) MammaPrint®, and (3) and no genomic profile testing. The model included chemotherapy costs, administration costs, short- and long-term adverse event costs, productivity loss, genomic profiling testing costs, cost of cancer recurrence, and hospitalization costs.

**Results:**

A treatment paradigm with Oncotype DX resulted in average savings per patient of €6,768 vs. a paradigm with MammaPrint and €13,125 vs. a paradigm with no genomic testing. Furthermore, due to less patients receiving adjuvant chemotherapy through better targeting by the Oncotype DX test, fewer adverse events, sick days, practice visits, and hospitalizations were required compared to MammaPrint and no genomic profiling.

**Conclusions:**

Testing with Oncotype DX test in Dutch clinical practice in patients with early-stage breast cancer proved to be cost-saving versus MammaPrint and no genomic profiling tests. Introducing the Oncotype DX test to the Dutch setting will likely reduce the economic resources that are required.

## 1. Introduction

Breast cancer is the most common type of cancer among women in the Netherlands, leading to approximately 17,000 incident cases and 3,000 fatalities per year [1]; in 2020; it accounted for 26.2% of new cancers in women of all ages [2]. For early-stage breast cancer, patients generally undergo surgical removal of the tumor. Although such surgery removes the primary tumor, most patients receive adjuvant systemic (chemo- and/or endocrine) therapy to prevent recurrence [3].

In recent years, genomic profile tests have become clinically accepted into practice guidelines; by analyzing tumor genomic expression, these tests can predict the prognosis of the tumor, such as the risk of future recurrence and for Oncotype DX, estimate the chemotherapy benefit. The Netherlands' breast cancer guidelines indicate that genomic profile tests could be helpful at identifying subgroups of patients that have different prognoses (favorable or unfavorable) [3]. Traditionally, clinicians use clinical factors including patient age, tumor size, grade, and lymph node status to make decisions regarding initiation of chemotherapy. These guidelines indicate that genomic profile tests can be used in cases where there is uncertainty regarding the benefit of adjuvant chemotherapy [3].

Adjuvant chemotherapy is associated with relatively high healthcare expenditures and toxicity for most patients. Furthermore, not all women benefit equally from it [4, 5]. In addition, chemotherapy is associated with a wide range of side effects, such as neutropenia and other blood disorders, alopecia, nausea, vomiting, gastrointestinal toxicities, fatigue, cardiotoxicity (e.g., anthracyclines) and neurotoxicity (e.g., taxanes). Given the clinical impact and high associated costs of adjuvant chemotherapy for patients and healthcare systems in general, diagnostic genomic profile tests have been developed to support evidence-based decision-making with respect to the benefit of adjuvant chemotherapy for individual patients.

Genomic profile testing provides multiple benefits. It can reduce the number of patients with lower-risk cancers receiving unnecessary aggressive treatments such as adjuvant chemotherapy, thereby reducing healthcare costs and avoiding toxicities without compromising patient outcomes. It provides information about risks and recurrence, so that clinicians are able to identify patients with high-risk disease that would benefit from adjuvant chemotherapy [6]. By allowing physicians and patients to make better informed decisions about initiating adjuvant chemotherapy, more individualized treatment plans can be created, and this substantially lessens the economic and clinical burden associated with unnecessary chemotherapy.

The Oncotype DX test is a multigene assay that quantitatively measures the expression of 21 genes (16 cancer-related genes and five reference genes) using reverse-transcriptase polymerase chain reaction (RT-PCR). The 16 cancer-related genes are associated with estrogen, proliferation, HER2, and invasion [7, 8]. The expression of the 16 oncogenes and five reference genes measured by the Oncotype DX test is used to calculate a Recurrence Score® result between 0 and 100, which can predict prognosis and whether adjuvant chemotherapy is of benefit for patients with early-stage HR+/HER2- N0 breast cancer [9–11]. A key clinical trial for Oncotype DX test is Trial Assigning Individualized Options for Treatment (TAILORx) [12], which included over 10,000 patients and focused on HR+ node negative early-stage breast cancer patients who obtain a midrange Recurrence Score® (11 to 25). The main objective of this trial was to determine whether patients with midrange scores would benefit from adjuvant chemotherapy. Patients were randomized into two treatment groups: those who received endocrine therapy alone and those who received endocrine therapy and chemotherapy. The results showed that endocrine therapy was not inferior to chemo-endocrine therapy in this cohort of patients with respect to invasive disease-free survival rates and overall survival. Therefore, patients with midrange scores from Oncotype DX test do not significantly benefit from the addition of adjuvant chemotherapy to endocrine therapy. Recently, the second large trial with Oncotype DX, RxPONDER [13] with approximately 5,000 patients, reported its first results for patients with micrometastasis, or up to 3 lymph nodes affected and demonstrated that postmenopausal patients with Recurrence Score results 0 to 25 did not benefit from chemotherapy.

Oncotype DX test has also been validated in several other clinical trials. The ability of the test to estimate the risk of recurrence was reported in the three clinical trials NSABP-B-14 [10], TransATAC [14], and Plan B [15]. In trials, NSABP B-20 [11, 16] and SWOG-8814 [9] Oncotype DX test showed that it could provide insight into the benefit of chemotherapy for each individual patient. Finally, the multinational prospective randomized trials TAILORx [12] and clinical trial Rx for Positive Node, Endocrine Responsive Breast Cancer (RxPONDER) [13] recently demonstrated that adjuvant chemotherapy can be safely omitted in node negative and node positive (1-3 nodes) women with early-stage hormone receptor positive and HER2 negative (HR+/HER2-) breast cancer and Recurrence Score results 0 to 25.

Oncotype DX test has already been used in the Rotterdam-based Breast Cancer Center South Holland South, resulting in fewer unnecessary and burdensome chemotherapy treatments and thus less toxicity by patients with early-stage breast cancer and was recently reimbursed by the Dutch health authorities. MammaPrint® is another genomic profile test only commercially available in the Netherlands. The latter is a microarray-based 70-gene signature test that classifies patients as low or high risk of distant recurrence [17–20]. Interestingly, there is an overlap of only 1 gene (SCUBA2) between the 21-gene and the 70-gene signature. The assay was validated in a heterogeneous population, including node-positive, node-negative, ER-positive, and ER-negative breast cancer patients treated with a variety of therapies [21]. The prognostic ability of MammaPrint has been demonstrated in clinical studies. However, the test has not been validated to predict chemotherapy benefit [18, 19, 22].

The aim of this study is to quantify the likely benefits that genomic profile tests can add for patients with early-stage HR+/HER2- node-negative (N0) breast cancer across the Netherlands. Therefore, a cost-consequence model was developed that compares two different types of genomic profile testing, Oncotype DX test and MammaPrint, with each other and with no genomic profile testing.

## 2. Materials and Methods

The cost-consequence model was designed to capture the relevant costs, resource use, and adverse events/sick days associated with chemotherapy following a treatment paradigm with Oncotype DX test, MammaPrint, and no genomic testing, to assess the impact of each test if implemented. The model considers the inputs from a societal perspective and for all costs. Regarding genomic test acquisition costs and drug costs, flat prices have been used; however, for all other costs (i.e., resource use), the prices retrieved have been inflated to reflect 2019 prices and are in the Euro (€) currency.

To represent the population as accurately as possible, the proportion of patients in the Netherlands with early HR+/HER2- N0 breast cancer who are eligible for chemotherapy was estimated as follows. Early HR+/HER2- N0 breast cancer is defined as those who have disease stage I or II HR+/HER2- N0 breast cancer. The number of women in the Netherlands that are diagnosed with invasive breast cancer annually was approximated from IKNL [23]. The proportion of patients who had disease stage I or II was sourced from IKNL and data from Oncotype IQ [23, 24].The proportion of patients who have HR+/HER2- N0 breast cancer was derived from the Nabon Breast Cancer Audit (NBCA) [25]. The Dutch national health institute estimated that approximately 30% of the patients with stage I or II HR+/HER2- N0 early breast cancer would be eligible for use of genomic tests [26]. The model considered only those patients with a clinical high risk.

**Figure 1:**
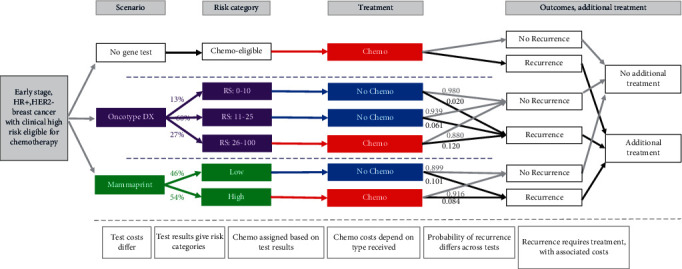
Diagram showing an overview of the structure for the cost-consequence model.

The model compares three scenarios: use of Oncotype DX test, use of MammaPrint, and no genomic test (current clinical practice in the Netherlands). A schematic overview of the model structure is shown below in Figure 1. The cost categories included in the model are costs associated with chemotherapy drug and drug administration, short- and long-term adverse events (AEs) associated with chemotherapy, productivity losses, acquisition of genomic profiling tests, and cancer recurrence.

For each scenario, associated costs were combined and totaled, accounting for the eligible population, distribution of risk scores per scenario, and the costs associated per cost category. There is functionality in the model to eliminate any of the cost categories discussed, with outcomes being adjusted accordingly. All inputs/calculated inputs used in the model alongside their sources are presented in Table 1.

Each paradigm—i.e., Oncotype DX test, MammaPrint, and no genomic test—was assigned a distribution of risk score that determined the proportion of patients that would receive adjuvant chemotherapy. For the results obtained from Oncotype DX test, patients were categorized into Recurrence Score results 0 to 10, RS 11 to 25, and RS 26 to 100. The MammaPrint test categorized patients into binary low and high risk; for no genomic testing, clinicians would categorize patients into low and high clinical risk based upon clinical and pathologic features according to the following: “clinical risk was defined as low if the tumor was 3 cm in diameter or smaller and had a low histologic grade, 2 cm or smaller and had an intermediate grade, or 1 cm or smaller and had a high grade; the clinical risk was defined as high if the low-risk criteria were not met” [12]. Patients with a tumor size of >50 mm were not considered early stage and as such were excluded. The model assumes that only high-risk patients are initiated on adjuvant chemotherapy and therefore these statistics form the fundamentals of the model.

The most frequently used chemotherapy regimens in early-stage breast cancer setting were included in the model and validated by a Dutch clinical expert. Regimens consisted of (1) three cycles of fluorouracil-epirubicin-cyclophosphamide followed by three cycles of docetaxel, (2) four cycles of doxorubicin-cyclophosphamide followed by 12 cycles of paclitaxel, and (3) four cycles of doxorubicin-cyclophosphamide followed by four cycles of docetaxel. The model assumed that four treatments of granulocyte colony–stimulating factor (G-CSF) would be given per chemotherapy regimen to mitigate neutropenia associated with chemotherapy; this has been sourced from Dutch clinical expert validation. The model assumed that 50% of patients who were initiated on adjuvant chemotherapy underwent regimen 1 and 25% of patients underwent regimens 2 and 3 each. These assumptions were validated by a Dutch clinical expert (a full list of assumptions is presented in Table 2). One of the main assumptions concerns adjuvant chemotherapy regimens and patient distributions. This data has been sourced from the medical literature as well as being validated by Dutch clinical experts and are likely to reflect clinical practice.

To calculate chemotherapy costs, the drug acquisition costs for each component of the regimens were sourced from *Z*-index in October 2020 [27]. The cost/milligram of each drug was calculated, and this was multiplied by the dose in milligrams per cycle to calculate the total cost per cycle. This was then multiplied by the total number of cycles in that regimen. These calculations were replicated across each regimen, and total costs were calculated. Costs associated with chemotherapy administration were calculated across the three regimens in a similar manner. The costs associated with chemotherapy administration, blood panel, and outpatient stay were multiplied by the frequency and the costs totaled. These were sourced from Dutch diagnosis-related group (DRG) tariffs [28] and Dutch manual for costing studies in health care [29]. In addition to this, port implantation costs were included for 10% of patients.

Costs relating to adverse events were split into short term and long term. The short-term adverse events included were neutropenia, myalgia and arthralgia, fatigue, infection, vomiting, nausea, neurosensory neuropathy, fever, diarrhea, constipation, dyspnea, stomatitis, anemia, skin rash, deep-vein thrombosis, thrombocytopenia, cough, superficial thrombophlebitis, and alopecia. Costs associated with grade I/II and III/IV events were obtained from the literature along with the proportions of patients experiencing both categories. For example, the proportion of patients experiencing grade I/II events for infection were multiplied by the costs associated with grade I/II infection. Long-term adverse events included in the model were acute myeloid leukemia and chronic heart failure. The 10-year probability of these two events occurring was obtained from the literature and multiplied by the relevant costs.

Productivity losses were calculated using the friction cost method [29]. Using national statistics, the percentage of females in the age bracket of 15-65 years with early-stage breast cancer who work in the Netherlands was calculated. The working hours per week were split into five categories: the proportion of patients who worked <12 hours/week, 12-20 hours/week, 20-28 hours/week, 28-35 hours/week, and full time. Using this data, the average number of hours was calculated and multiplied by the average productivity cost/hour and was corrected for the retirement age.

Productivity costs are based on assumptions regarding the percentage of women who are employed, duration of sick leave, and those who are <65 years (retirement age).

Genomic profile testing acquisition costs for Oncotype DX test and MammaPrint were also included. The 10-year probability of recurrence with chemotherapy was calculated per risk group across all three scenarios. These probabilities were then multiplied by the mean cost per patient for recurrence. Cost of hospitalization was sourced from the Dutch costing manual [29]. The 10-year probability of recurrence with chemotherapy between Oncotype DX test and no genomic test was assumed to be similar, leading to similar costs.

## 3. Results

Overall, it was estimated that 15,000 patients would be diagnosed with early invasive breast cancer annually. Of these, 73% are estimated to have either disease stage I or II. Proportions of patients who have HR+/HER2- mutation and who were N0 were 74.7% and 82.8%, respectively. Of these, 30% of patients would be considered eligible for genomic testing. This led to a total model population of 2,032 patients. The results generated are broken down into costs per cost category and total costs per patient per test or no testing. A breakdown of all the costs can be found in Table 3. For all cost categories, the model (aside from the acquisition costs of genomic testing) predicts that the two genomic profile tests are cost-saving compared with no genomic testing. Oncotype DX test is predicted to be less costly compared with both MammaPrint and no genomic test. Overall, the results show that Oncotype DX test is cost saving compared with both other practices.

### 3.1. Adjuvant Chemotherapy and Administration Costs

For the entire model population, the results show that MammaPrint is €13,750,478 more costly than Oncotype DX test, and that no genomic testing is more costly than Oncotype DX test by €26,667,347. This results in per patient savings of €6,768 and €13,125, respectively. In total, the model predicts that there are 549 fewer adjuvant chemotherapies initiated when Oncotype DX test is used compared with MammaPrint and 1,483 fewer chemotherapy initiations compared with no genomic testing. These predictions are based largely on the distribution of the risk scores that were assigned to each scenario (as detailed in Table 1). This is reflected in the results, as fewer patients would need chemotherapy in the Oncotype DX test intervention, resulting in cost savings and further added benefit for patients. Consequently, with fewer patients receiving chemotherapy, the associated administration costs are smaller in the Oncotype DX test intervention in comparison with the MammaPrint intervention and no genomic testing. The savings relating to adjuvant chemotherapy and administration costs are predicted to be €7,857,631 when Oncotype DX test is compared against MammaPrint and €21,244,707 when Oncotype DX test is compared against no genomic testing. Per patient, this equates to savings of €3,867 and €10,455, respectively.

### 3.2. Adverse Events

The cost savings from Oncotype DX test are associated with the reduction in short-term adverse events between Oncotype DX test versus MammaPrint, and no genomic testing for the entire patient population is €1,492,789 and €4,036,059, respectively, and per patient is €735 and €1,986, respectively. With fewer patients receiving chemotherapy, fewer patients experience adverse events associated with chemotherapy, such as neutropenia, fatigue, and anemia. A similar outcome is seen with the costs of long-term adverse events where use of Oncotype DX test is estimated to save €563,146 in comparison to MammaPrint and €1,522,580 compared to no genomic testing. Per patient, this equates to a saving of €277 and €749, respectively. It is predicted that overall, 505 short- and long-term adverse events are avoided when Oncotype DX test is used compared to MammaPrint and 1,364 adverse events are avoided when compared to no genomic testing.  Table 4 outlines outcomes relating to adverse events generated by the model and hospital visits relating to adverse events.

### 3.3. Productivity Losses

Comparing the three interventions, the model predicts that costs associated with productivity losses are the least with use of Oncotype DX test. The cost of productivity losses is €3,321,681 less with Oncotype DX test than MammaPrint and €8,980,842 less than no genomic testing. Per patient, Oncotype DX test saves €1,635 than MammaPrint and €4,420 compared to no genomic testing. This is expected, as it is likely that fewer patients experience sick days when they do not receive chemotherapy. It is estimated that overall, there are 61,048 fewer sick days when Oncotype DX test is implemented and 4,937 fewer practice visits compared to MammaPrint, and 165,055 fewer sick days with 13,349 fewer practice visits compared to no genomic testing.

### 3.4. Disease Recurrence

Cost of disease recurrence is less for Oncotype DX test compared to MammaPrint by €1,965,020 and was assumed similar compared to no genomic testing. Per patient, this equates to €967 for MammaPrint.

### 3.5. Oncotype DX Test vs. MammaPrint

Although the two genomic profile tests are cost-saving by reducing chemotherapy utilization compared to no genomic testing, costs for Oncotype DX test are lower than for MammaPrint across all cost categories apart from acquisition cost. The acquisition cost of Oncotype DX test is higher than that of MammaPrint and for the entire model population, Oncotype DX test is more expensive by €1,449,788 and €713 per patient. However, the net saving for Oncotype DX test is greater as per patient; Oncotype DX test saves €6,768 in comparison to MammaPrint and €13,750,478 across the entire model population. The clinical burden on patients is reduced with Oncotype DX test as fewer patients are likely to receive chemotherapy and the consequent associated costs are lessened.

### 3.6. Oncotype vs. No Genomic Testing

The Oncotype DX test also saves cost (vs. no genomic testing) in all categories aside from acquisition costs. Oncotype DX test saves €31,314,652 compared to no genomic test and saves €15,412 per patient.

## 4. Discussion

The results from the cost-consequence model indicate that implementing genomic profiling testing in clinical practice in the Netherlands can have immense financial savings for the Dutch healthcare system, especially with Oncotype DX test. The fundamental prediction behind the considerable savings is that Oncotype DX test will prevent a greater number of patients from undergoing unnecessary adjuvant chemotherapy compared to both MammaPrint and no genomic test in early-stage breast cancer. This is based on the distribution of risk scores retrieved from the TAILORx [12] and MINDACT clinical trials [40]. The distribution of risk scores assigned to each scenario is based on clinical trial studies for the genomic profile tests and IKNL data for no genomic testing; these risk scores are likely to be representative of true distributions in practice. For example, a study in France [43] showed that Oncotype DX test reduced the number of patients receiving chemotherapy by 23% and recommended initiating in 11% of patients who were originally commenced on endocrine therapy alone. Similar results are seen in a study conducted with Spanish patients [44].

Another study conducted in Spain was designed to assess the clinical and economic impact of Oncotype DX and MammaPrint compared to Spanish standard practice. The population in the study was patients with ER+/HER2-, N0 or micrometastatic stage I or II breast cancer. The study found that using the two genomic tests to assess the benefit of adjuvant chemotherapy in these patients resulted in many patients being spared chemotherapy and overall was cost-effective [45]. The study reports that when Oncotype DX test was used, adjuvant therapy for 34.5% of patients was changed from chemohormonal therapy to hormonal therapy only [45]. When MammaPrint was used, adjuvant therapy for 26.8% of patients was changed from chemohormonal therapy to hormonal therapy only [45]. The outcomes from the study show that there were more patients who did not undergo adjuvant chemotherapy when Oncotype DX was used compared to MammaPrint; this is also reflected in the cost-consequence model built.

Previous economic studies suggest that Oncotype DX test is cost-effective. Holt et al. discuss a cost-effectiveness model that was built comparing Oncotype DX test testing with usual care (no testing) in the UK to see the differences in the proportion of patients who were prescribed chemotherapy and the difference in quality of life and costs between the two. The study found that using Oncotype DX test, chemotherapy for 26 out of 57 patients (45.6%) was not prescribed, and instead, these patients received only hormonal therapy, and 12 out of 85 patients (14.1%) received advice based on Oncotype DX test results to commence chemotherapy [46]. It was estimated that the use of Oncotype DX test resulted in £6,232 per quality-adjusted life-year and £5,633 per life-year gained [46]. Change in baseline age and change in chemotherapy use were biggest key drivers of the model. The study also reports that genomic profile testing increased patients' confidence regarding their treatment and decisions around it [46]. Similar results of Oncotype DX test being cost-effective/cost-saving compared to no genomic testing are seen in studies focusing on patients in the United States [47], Canada [48], Germany [49], and Israel [50].

A key driver of the cost-consequence model is the proportion of patients being initiated on adjuvant chemotherapy; such initiation leads to a cascade of clinical consequences for patients and associated costs. Notably, monitoring and follow-up costs have not been incorporated into the model due to the assumption that these would be the same regardless of whether genomic testing is implemented and therefore would cancel one another out.

The costs associated with disease recurrence constitute another key driver of the cost differences between the interventions. To assess the impact, these recurrence costs were eliminated from the model, and the outcomes were adjusted accordingly. A similar trend in results was shown with Oncotype DX test saving costs in all cost categories aside from acquisition costs compared with MammaPrint. Without considering recurrence costs, Oncotype DX test is predicted to be €5,800 less expensive per patient than MammaPrint. In addition to this, there are other important added benefits of implementing Oncotype DX test. The model predicts that 1,483 fewer patients will be initiated on chemotherapy with chemotherapy cost savings of €21,244,707 in the entire model population (€10,455 per patient). In addition to this, similar to the base case results, use of Oncotype DX test results in fewer chemotherapy costs, adverse events, hospitalizations, and productivity losses.

With the model estimating 1,483 patients being overtreated with adjuvant chemotherapy, it gives rise to discussions to how the allocation for the resources used for these patients can be optimized. For example, the vials of chemotherapy used for these additional 1,483 patients incur unnecessary costs as they provide minimal benefit for these patients. However, using the additional information provided by Oncotype DX test, the chemotherapy agents can be used with patients who would likely benefit from them. This will lead to optimized supply of chemotherapy treatment for those patients in early-stage breast cancer and across other disease areas where these chemotherapies are used. With more optimized use of chemotherapy, there is reduced financial burden on the Netherlands' healthcare system and reduction in chemotherapy burden on patients who would not derive benefit from it. Diagnostic testing via genomic profiling tests is becoming more and more important as it might inform and support physicians in decision-making, thereby providing the best available treatment for the patient. Additionally, the cost savings that are generated, due to better allocation of treatment and reduced adjuvant chemotherapy usage, can be used more efficiently for other healthcare purposes but also frees up time for physicians and healthcare providers to help other patients. Next to that, it can help payers understand the need for genomic testing, and although costs might be spent upfront, using a test such as Oncotype DX eventually leads to financial benefits in a 6-month to 10-year timeframe.

A reduction in chemotherapy initiation will inevitably result in far fewer adverse events occurring and therefore fewer hospital visits for patients. Short-term adverse events usually occur during chemotherapy and shortly after completion while long-term events can occur much later after chemotherapy treatment and potentially last for a longer period of time [4]. Neutropenia is a common adverse event associated with many types of chemotherapies. Treatment for this is typically G-CSF administration during chemotherapy cycles and can sometimes result in patients requiring hospital visits. Adverse events like this directly impact the Netherlands' healthcare system and result in additional financial expenditure. Other adverse events such as fatigue and weight gain—although they do not necessarily impact the healthcare system directly—have an impact on patients' health-related quality of life and can affect their daily activities, as well as their mental health [51].

Similarly, long-term adverse events associated with chemotherapy can be both a financial burden on the healthcare system and a clinical burden on patients. Doxorubicin and epirubicin are used commonly in the Netherlands for early-stage breast cancer and form part of the three chemotherapy regimens typically used and are included in the model. These chemotherapy regimens are associated with cardiotoxicity, such that when patients are exposed to these treatments, their risk of cardiac dysfunction and heart failure increases [4]. Long-term complications like cardiotoxicity result in further health deterioration for patients, reducing their quality of life and adding pressure on the healthcare system financially to treat and manage these effects.

Consequently, patients experiencing adverse events from adjuvant chemotherapy are likely to have reduced productivity. Generally, time off work may be necessary to attend hospital appointments or undergo chemotherapy administration. However, patients experiencing fatigue, gastrointestinal effects, and other adverse events may not feel well enough to work, resulting in productivity losses that can have wide effects on patients themselves, their employers, and the economy. Employers may need to recruit and train new staff to maintain services while employees are off sick, which can result in more expenditures. Across different factors and perspectives, productivity losses can incur substantial expenses.

Usage of genomic profiling tests such as Oncotype DX test in routine clinical practice can ease much of this financial and clinical burden. The impact that can occur from introducing Oncotype DX test to clinical practice is substantial across different areas. The additional information regarding disease recurrence and the benefit of adjuvant chemotherapy for each patient will allow for a reduction in the number of patients receiving chemotherapy itself, thereby reducing how many experiencing associated adverse events and productivity losses.

## 5. Limitations

One limitation of the model is the use of different sources in calculating the 10-year disease recurrence probabilities. Due to limited data on this, the TAILORx [12] and MINDACT [18] clinical trial data was used to inform the recurrence probabilities with use of Oncotype DX and MammaPrint, respectively. For Oncotype DX, the nine-year reported probability for recurrence was adjusted to reflect the 10-year probability, and for MammaPrint, the five-year reported probability was adjusted also to reflect 10 years. Regarding no genomic testing, due to the lack of 10-year recurrence data, the documented Oncotype DX recurrence was used to inform the 10-year recurrence rate instead. Using multiple sources is likely to result in the analysis of the different interventions being less comparable.

Finally, the model assumes that for the no genomic testing scenario, all patients who are considered clinically high risk will receive adjuvant chemotherapy. Although in real life most patients would be commenced on adjuvant chemotherapy, there would be some patients where chemotherapy would be contraindicated. For example, some patients may have other comorbidities where chemotherapy is unsuitable for them.

## 6. Conclusions

Overall, the use of the genomic profiling test Oncotype DX test in patients with early-stage HR+/HER2- N0 breast cancer is shown in a cost-consequence economic model to have significant impacts on patients in this population and substantial cost savings for the healthcare system in the Netherlands. The outcomes of the model suggest that Oncotype DX test can identify and reduce the number of patients receiving adjuvant chemotherapy, as well as identifying those who may have otherwise been undertreated. Overall, Oncotype DX test saves greater costs compared with MammaPrint and no genomic testing. By reducing the number of patients receiving adjuvant chemotherapy, the use of Oncotype DX test results in fewer patients experiencing AEs associated with chemotherapy. Implementing Oncotype DX test at a national level can alleviate the pressure and financial burden on the Netherlands' healthcare system, while minimizing the number of patients exposed to chemotherapy without compromising patient health.

## Figures and Tables

**Table 1:** The list of inputs/calculated inputs used in the cost-consequence model and their sources.

Parameter	Subparameter	Value	Source
Patient population	Approximate number of patients in the Netherlands diagnosed with invasive breast cancer annually	15,000	Incidence Breast Cancer IKNL [23]
	Proportion of patients with disease stage I or II	73%	IKNL and Oncotype [23, 24]
	Proportion of patients with HR+/HER2 early-stage invasive breast cancer	74.7%	NBCA [25]
	Proportion of patients with N0 early-stage invasive breast cancer	82.8%	NBCA [25]
	Proportion of patients who would be considered eligible for genomic testing	30%	ZIN viewpoint Oncotype [26]
	Mean body surface area (m^2^)	1.75	

Distribution of risk score result	Oncotype DX test—RS 26 to 100	27%	Sparano [12]
	Oncotype DX test—RS 11 to 25	60%	Sparano [12]
	Oncotype DX test—RS 0 to 10	13%	Sparano [12]
	MammaPrint—low risk	46%	Cardoso [18]
	MammaPrint—high risk	54%	Cardoso [18]
	No genomic test—low risk	0%	Dutch clinical expert
	No genomic test—high risk	100%	Dutch clinical expert, only patients that are eligible for chemotherapy are provided a genomic test as part of the Dutch clinical setting

General	Average number of supportive G-CSF rounds given per treatment regimen	4	Dutch clinical expert

Distribution of chemotherapy regiments	3xFEC100+3xT	50%	Dutch clinical expert Retèl [30]
	4x(dd)AC+12xP (wkl)	25%	
	4x(dd)AC+4xT	25%	

Total chemotherapy regimen acquisition costs	3xFEC100+3xT	€5723.67	*Z*-Index [27]
	4x(dd)AC+12xP (wkl)	€6966.36	
	4x(dd)AC+4xT	€5964.17	

Chemotherapy administration costs per regimen	3xFEC100+3xT	€5428.13	DRG: code 020107015 [28] DRG tariff table: code 070201 [31] [29]
	4x(dd)AC+12xP (wkl)	€14,407.52	
	4x(dd)AC+4xT	€7230.01	

Port implantation	Occurs in 10% of patients	€105.34	Dutch DRG tariff table

Short-term AE costs	Total grade I/II	€444.31	Bouwmans [32], Wehler [33], ZIN assessment palbociclib (Ibrance®), ZIN assessment evrolimus (Afinitor) [34, 35]
	Total grade III	€1991.77	
	Total grade IV	€287.15	

Long-term AE costs	Acute myeloid leukemia	€667.31	Wolff [36], Leunis [37], ZIN reassessment trastuzumab (Herceptin), Boekel [38]
	Chronic heart failure	€359.22	

Productivity losses	% gross labor participation (15-65 years)	74%	Dutch costing manual [29]
	Average worked hours per person per week corrected for labor rate	20	
	Proportion of early-stage breast cancer patients below retirement age	56.45%	
	Productivity cost per hour, women	€33.75	
	Productivity costs per day, corrected for proportion above retirement age	€54.41	
	Friction period (days)	111	
	Total productivity loss per person	€6054.91	

GPT costs	Oncotype DX test	€4487.02	Maximum tariff for Oncotype DX test and MammaPrint. NZA: ZA-code 050531 for Oncotype DX test and NZA: ZA code 050530 for MammaPrint, 2021
	MammaPrint	€3773.48	

Cost of recurrence	Mean cost per patient for recurrence	€47,211.28	Thomas [39]

10-year probability of distant recurrence with chemotherapy	Oncotype DX test RS 0 to 10	0.020	Sparano [12], Cardoso [40], Harnan [41] Dutch clinical expert
	Oncotype DX test RS 11 to 25	0.061	
	Oncotype DX test RS 26 to 100	0.120	
	MammaPrint low	0.101	
	MammaPrint high	0.084	
	No genomic test low	Costs assumed to be similar to Oncotype DX test	
	No genomic test high		

Cost of hospitalization	—	€605.32	Dutch costing manual [29]

Abbreviations: FEC = fluorouracil-epirubicin-cyclophosphamide; T = docetaxel; (dd)AC = doxorubicin-cyclophosphamide; P = paclitaxel; G-CSF = granulocyte colony-stimulating factor; AEs = adverse events; RS = recurrence score.

**Table 2:** The full list of assumptions used in the cost-consequence model.

Assumption	Content	Source
BSA	1.75 m^2^	Dutch clinical expert validation
Vial sharing	Vial sharing assumed	Assumption
Population	The model assumes that only clinically high-risk patients will be populated in the model. It is not Dutch common practice for genomic testing to be used in patients with low clinical risk	Assumption
Monitoring/follow-up	It was assumed that monitoring/follow-up visits were the same in both treatment arms (no adjuvant/adjuvant chemotherapy) regardless of treatment with adjuvant chemotherapy and irrespective of the type of testing. Hence, these costs were not included as they balance each other out	Medical team exact sciences
Endocrine therapy	It was assumed that endocrine therapy was similar irrespective of whether GPT testing was used; thus, these costs were not included	Assumption
Survival	Long-term or short-term survival outcomes are not taken into consideration in this analysis	Assumption
Productivity costs	(i) Percentage of patients < 65 years derived from IKNL (ii) Percentage of women employed derived from Statistics Netherlands (CBS) (iii) Sick leave duration and full-time work derived from Lux et al. (2017)	Percentage of patients < 65 years derived from IKNL Lux [42]
Hospitalizations due to short-term AEs	Hospitalization due to grade III/IV AEs were not considered as the cost associated with each individual AE is already implemented in the model	Dutch clinical expert opinion
Grade I/II short-term AEs (except alopecia)	It was assumed that 10% of grade I/II AEs required an outpatient visit	Dutch clinical expert opinion
Alopecia	It was assumed that 100% of patients treated with chemotherapy for early-stage breast cancer developed grade II (= total) alopecia unless treated with a cold cap. About 50% of patients chose a cold cap during chemotherapy, protecting against total alopecia in about half of them. All patients get a prescription for a wig before starting chemotherapy (irrespective of being treated with a cold cap or not). Hence, alopecia costs are calculated as follows: 100% insurance coverage cost for a wig + 50% cold cap costs	Dutch clinical expert opinion

Abbreviations: AEs = adverse events; IKNL = Integraal Kankercentrum Nederland; GPT = genomic profile test.

**Table 3:** Results presented for each cost category across all three scenarios. Total costs per patient are calculated, and all results are in Euros.

Clinical practice	Chemotherapy acquisition costs (€)	Chemotherapy administration costs (€)	Short-term AE costs (€)	Long-term AE costs (€)	Productivity losses (€)	GPT test costs (€)	Recurrence costs (€)	Total costs for entire model population (€)	Total costs per patient (€)
Oncotype DX test	€3,343,380	€4,514,251	€1,492,789	€563,146	€3,321,681	€9,116,840	€6,863,702	€29,215,789	€14,379
No genomic test	€12,382,889	€16,719,449	€5,528,848	€2,085,726	€12,302,523	€0	€6,863,702	€55,883,137	€27,504
MammaPrint	€6,686,760	€9,028,502	€2,985,578	€1,126,292	€6,643,362	€7,667,051	€8,828,722	€42,966,268	€21,147
Difference between Oncotype DX test vs. no genomic test	-€9,039,509	-€12,205,198	-€4,036,059	-€1,522,580	-€8,980,842	€9,116,840	€0	-€26,667,347	-€13,125
Difference between Oncotype DX test vs. MammaPrint	-€3,343,380	-€4,514,251	-€1,492,789	-€563,146	-€3,321,681	€1,449,788	-€1,965,020	-€13,750,478	-€6,768

Abbreviations: AE = adverse event; GPT = genomic profile test.

**Table 4:** Results presenting outpatient chemotherapy visits and data relating to AEs and hospital visits relating to AEs.

	Outpatient visits		Hospitalizations, outpatient visits, and number of total AEs	
Clinical practice	Outpatient chemotherapy visits total	Hospitalizations due to short-term grade III/IVs AEs total	Outpatient visits due to short-term AE total	Number of AE total (short term and long term)
Oncotype DX test	4,937	97	482	505
No genomic test	18,286	361	1,784	1,869
MammaPrint	9,875	195	964	1,009
Difference between Oncotype DX test vs. no genomic test	-13,349	-264	-1,303	-1,364
Difference between Oncotype DX test vs. MammaPrint	-4,937	-97	-482	-505

Abbreviation: AEs = adverse events.

## Data Availability

The input data supporting this cost-consequence model are from previously reported publications and reports, which have been cited. No datasets were generated or analyzed during the current study.
